# Blockade of Orexin Receptors in the Posterior Paraventricular Nucleus of the Thalamus Prevents Stress-Induced Reinstatement of Reward-Seeking Behavior in Rats With a History of Ethanol Dependence

**DOI:** 10.3389/fnint.2020.599710

**Published:** 2020-11-10

**Authors:** Alessandra Matzeu, Rémi Martin-Fardon

**Affiliations:** Department of Molecular Medicine, The Scripps Research Institute, La Jolla, CA, United States

**Keywords:** ethanol, palatable food, dependence, orexin, TCS1102, pPVT

## Abstract

Neural systems involved in processing natural rewards and drugs of abuse overlap and exposure to drugs of abuse induce neuroadaptations that can cause compulsive-like behavior. For example, the recruitment of the orexin (Orx) system by drugs of abuse has been proposed to induce neuroadaptations that in turn alter its function, reflected by maladaptive, compulsive, and addictive behavior. Orexin neurons project to the paraventricular nucleus of the thalamus (PVT)—particularly the posterior part (pPVT), a structure that plays a key role in stress regulation. This study investigated whether Orx transmission in the pPVT plays a role in stress-induced reinstatement of reward-seeking behavior toward ethanol (EtOH) and a highly palatable food reward [sweetened condensed milk (SCM)] in rats and whether this role changes with EtOH dependence. After being trained to orally self-administer EtOH or SCM, the rats were made dependent (EtOH_D_ and SCM_D_) by chronic intermittent EtOH vapor exposure. The control nondependent groups (EtOH_ND_ and SCM_ND_) were exposed to air. Following extinction, the rats were tested for stress-induced reinstatement of EtOH- and SCM-seeking behavior. Stress reinstated EtOH- and SCM-seeking behavior in all groups (EtOH_D/ND_ and SCM_D/ND_). Administration of the dual Orx receptor (OrxR) antagonist TCS1102 (15 μg) in the pPVT prevented stress-induced reinstatement only in dependent rats (EtOH_D_ and SCM_D_). In parallel, the qPCR analysis showed that *Orx* mRNA expression in the hypothalamus and *OrxR1*/*R2* mRNA expression in the pPVT were increased at the time of testing in the EtOH_D_ and SCM_D_ groups. These results are the first to implicate Orx transmission in the pPVT in the stress-induced reinstatement of reward-seeking behavior in EtOH dependent rats and indicate the maladaptive recruitment of Orx transmission in the pPVT by EtOH dependence.

## Introduction

Drugs neuroadaptively influence neural systems that regulate motivation that is normally directed toward natural rewards. The neuroplasticity of this circuitry may be responsible for maladaptive compulsive behavior that characterizes addiction (e.g., Kelley and Berridge, 2002; Aston-Jones and Harris, 2004; Kalivas and O’Brien, 2008; Wanat et al., 2009). Relapse vulnerability is a challenge for the successful treatment of ethanol (EtOH) addiction, and relapse prevention has emerged as a major problem for treatment and medication development efforts (DeJong, 1994; O’Brien and McLellan, 1996). In patients who suffer from alcohol use disorder (AUD), relapse is frequently triggered by stressful events (Breese et al., 2005; Sinha, 2007). Over recent decades, behavioral scientists have used rodent models of reinstatement to study neuronal mechanisms of stress-induced relapse to EtOH seeking (Weiss et al., 2001; Lê and Shaham, 2002; Shaham et al., 2003) and investigate brain mechanisms that regulate palatable food-seeking behavior (Ghitza et al., 2006; Nair et al., 2009). The reinstatement of EtOH seeking following intermittent footshock has been extensively used to mimic relapse-like behavior in rodents (e.g., Lê et al., 1998, 1999; Martin-Fardon et al., 2000; Liu and Weiss, 2002), demonstrating that stress-induced relapse is a valid model for testing possible therapeutic targets for the prevention of craving and relapse.

The orexin (Orx; also known as hypocretin) system (de Lecea et al., 1998; Peyron et al., 1998; Sakurai et al., 1998) regulates physiological functions (Sutcliffe and de Lecea, 2000; Mieda and Yanagisawa, 2002; de Lecea, 2012), modulates stress (Berridge et al., 2010), and has been implicated in reward-motivated (e.g., drug-seeking) behavior (Harris et al., 2005; Dayas et al., 2008; Martin-Fardon et al., 2010, 2018; Jupp et al., 2011b; Sakurai and Mieda, 2011). Compulsive EtOH drinking is driven by dysfunctional reward and stress systems (Tunstall et al., 2017). Based on its role in regulating reward-motivated behavior and stress-related behaviors, the Orx system may be an ideal target for AUD treatment. The Orx system is recruited by both conventional reinforcers (Cason and Aston-Jones, 2013a,b; Olney et al., 2015) and drugs of abuse, including EtOH (Borgland et al., 2006; Bonci and Borgland, 2009; Thompson and Borgland, 2011). Orexin neurons are activated by stimuli that are predictive of food, morphine, cocaine, and EtOH (Harris et al., 2005; Dayas et al., 2008; Martin-Fardon et al., 2010, 2018; Jupp et al., 2011b). Pharmacological manipulations of the Orx system affect EtOH intake and seeking. For example, the OrxR1 antagonist SB334867 decreased voluntary home-cage EtOH drinking (Moorman and Aston-Jones, 2009; Anderson et al., 2014), operant EtOH self-administration (Lawrence et al., 2006; Richards et al., 2008; Jupp et al., 2011a; Lei et al., 2016a; Moorman et al., 2017), and the reinstatement of EtOH seeking that is induced by EtOH-associated stimuli (Lawrence et al., 2006; Jupp et al., 2011b; Martin-Fardon and Weiss, 2014; Brown et al., 2016; Moorman et al., 2017) or stress (Richards et al., 2008). SB334867 treatment disrupted EtOH-induced conditioned place preference (CPP) and EtOH-induced locomotor sensitization (Voorhees and Cunningham, 2011; Macedo et al., 2013). A few studies also showed that OrxR2 blockade with specific antagonists affected EtOH consumption. For example, LSN2424100 decreased home-cage EtOH drinking (Anderson et al., 2014), JNJ10397049 reduced EtOH self-administration in rats and EtOH-induced CPP and the reinstatement of operant responding for EtOH in mice (Shoblock et al., 2011), while an intracerebroventricular or intra-paraventricular nucleus of the thalamus (PVT) injection of TCSOX229 reduced EtOH intake in rats (Brown et al., 2013; Barson et al., 2015). These results suggest that the Orx system plays a key role in EtOH intake and EtOH-seeking behavior.

The PVT is part of dorsal midline thalamic nuclei and plays a key role in energy homeostasis, arousal, endocrine regulation, reward (Bhatnagar and Dallman, 1998; Van der Werf et al., 2002; Kelley et al., 2005; Parsons et al., 2006), and particularly stress regulation (Hsu et al., 2014). The effects of EtOH abuse on the thalamus have been recognized for decades. For example, the thalamus was shown to undergo marked volume reductions that resulted in characteristic cognitive impairments in severe alcoholics (Mann et al., 1999, 2001; Tedstone and Coyle, 2004; Pitel et al., 2015), and exposure to EtOH-related cues significantly activated the thalamus (George et al., 2001). Shrinkage of the thalamus was also observed in rats that were bred to consume high amounts of EtOH, suggesting that the predisposition to EtOH dependence in these rats may be associated with thalamic abnormalities that are reminiscent of those that are observed in EtOH-dependent patients (Gozzi et al., 2013). Among its extensive projections, Orx neurons send dense projections to the PVT, especially its posterior part (pPVT; Peyron et al., 1998; Baldo et al., 2003; Kirouac et al., 2005; Hsu and Price, 2009). The PVT is consistently and potently activated in rodents by various stressors and plays a unique role in regulating responses to chronic stressors (Hsu et al., 2014). For example, endogenous Orx release acted on the PVT to produce anxiety, and OrxR blockade in the PVT attenuated the anxiogenic effects of footshock stress (Li et al., 2010) and decreased the latency to engage in social interaction in a contextual fear conditioning paradigm in rats (Dong et al., 2015). Moreover, OrxA administration in the pPVT reinstated cocaine- and sweetened condensed milk (SCM)-seeking behavior (Matzeu et al., 2016, 2018), further supporting a pivotal function for Orx transmission in the pPVT in the mediation of reward-seeking behavior. A role for Orx projections to the PVT in EtOH seeking is supported by previous findings that EtOH-related contextual cues increased the number of Fos-positive PVT neurons that were closely associated with Orx fibers (Dayas et al., 2008). Other evidence supports a role for Orx projections to the PVT during EtOH seeking. The context-induced reinstatement of alcoholic beer seeking was associated with PVT–ventral striatum pathway recruitment, and inactivation of the PVT prevented the context-induced reinstatement of EtOH seeking (Hamlin et al., 2009; Marchant et al., 2010).

The pPVT receives the densest Orx projections (Kirouac et al., 2005). Based on our data on cocaine- and SCM- seeking behavior (Matzeu et al., 2016, 2018), the present study investigated whether the pharmacological manipulation of Orx transmission in the pPVT (i.e., OrxR1 and OrxR2 blockade with the dual Orx receptor antagonist TCS1102) prevents the stress-induced reinstatement of reward-seeking behavior toward EtOH and a highly palatable food reward (i.e., SCM) in rats with a history of EtOH dependence. To assess whether molecular changes that occur during EtOH dependence could explain the pharmacological results, we measured *Orx* mRNA expression in the hypothalamus and *OrxR1/R2* mRNA expression in the pPVT at the time of the reinstatement test. Overall, we tested the hypothesis that actions of Orx in the pPVT are important during stress-induced reinstatement of reward-seeking behavior following EtOH dependence.

## Materials and Methods

### Rats

One hundred eighty adult Wistar rats (90 males and 90 females; Charles River, Wilmington, MA, USA), 2 months old at the start of the experiment, were housed two per cage in a temperature- and humidity-controlled vivarium on a reverse 12 h/12 h light/dark cycle with *ad libitum* access to food and water. The animals were given at least 1 week to acclimate to the housing conditions and handling daily before testing. All of the procedures were conducted in strict adherence to the National Institutes of Health *Guide for the Care and Use of Laboratory Animals* and were approved by the Institutional Animal Care and Use Committee of The Scripps Research Institute. Both males and females were included in the study, not to study sex differences but rather to be inclusive of both sexes. Consequently, as shown in Figures 3, 4, males and females were not evenly distributed across the different groups.

**Figure 1:**
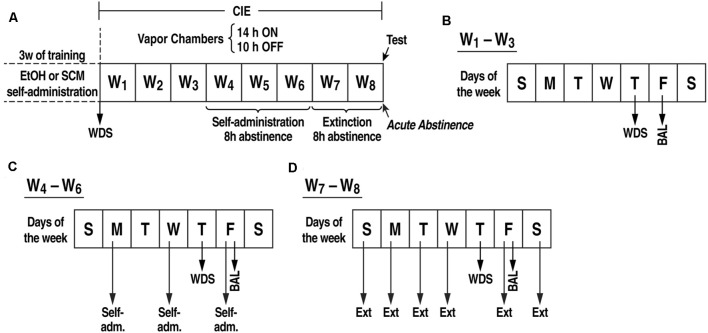
**(A)** Experimental procedure. Somatic withdrawal sign (WDS) scores were recorded upon the completion of training. **(B)** During weeks 1–3 of CIE vapor exposure, BALs were measured 15 min before the EtOH vapors were turned off (on Fridays), and the rats were scored for WDS 8 h (i.e., acute abstinence) after the EtOH vapor was turned off (on Thursdays). **(C)** During weeks 4–6 of CIE vapor exposure, the rats underwent self-administration sessions (Monday, Wednesday, and Friday) when acute abstinence occurred (8 h after the vapor was turned off). **(D)** During weeks 7–8 of CIE vapor exposure, the rats were exposed to daily extinction sessions during acute abstinence (8 h after the EtOH vapor was turned off). BAL, blood alcohol level; WDS, somatic withdrawal signs; W, week.

**Figure 2:**
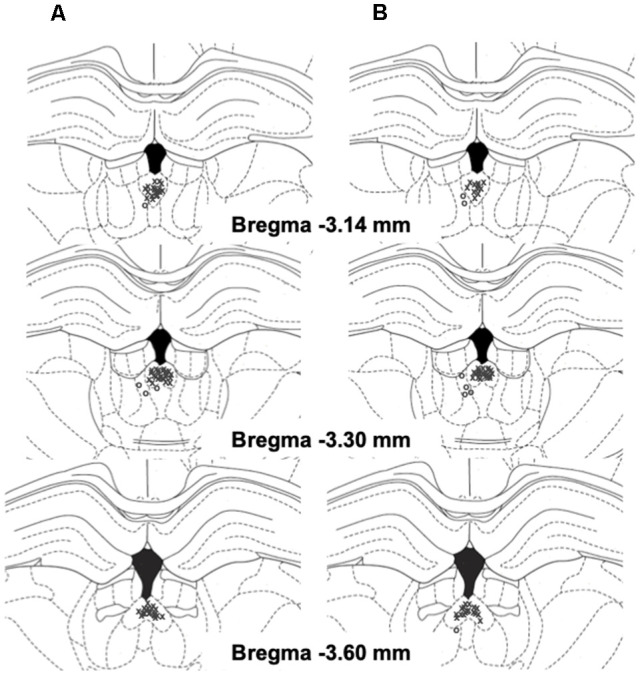
Schematic representation of injection sites in the pPVT (x, rats with correct injection sites; o, rats with missed injection sites) in EtOH **(A)** and SCM **(B)** rats.

**Figure 3:**
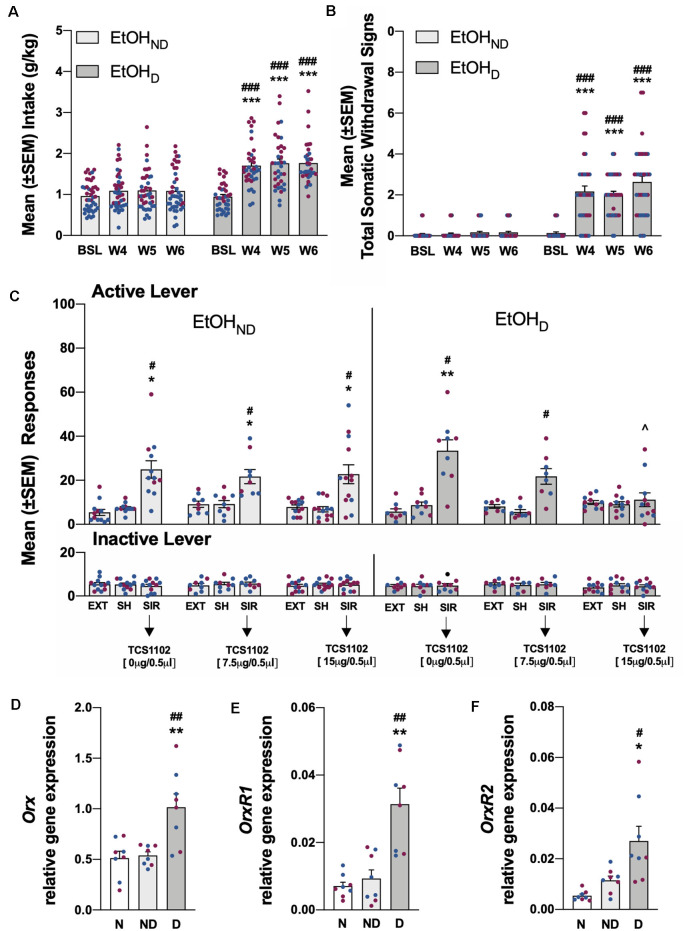
**(A)** Total EtOH intake in the EtOH_ND_ and EtOH_D_ groups during training and the air (EtOH_ND_) and CIE (EtOH_D_) vapor procedure. ***p < 0.001, vs. training; ^###^*p* < 0.001, vs. EtOH_ND_. *n* = 36–42 rats/group. **(B)** Somatic withdrawal signs (WDS) recorded upon the completion of training and during acute abstinence after CIE vapor exposure. ***p < 0.001, vs. baseline; ^###^*p* < 0.001, vs. EtOH_ND_. *n* = 36–42 rats/group. **(C)** Effect of TCS1102 injection in the pPVT on footshock stress-induced reinstatement of EtOH-seeking behavior. *p < 0.05, **p < 0.01, vs. respective extinction and sham injection; ^∧^*p* < 0.05, vs. respective vehicle; ^#^*p* < 0.05, vs. respective inactive lever. *n* = 8–13 rats/group. **(D)** Relative *Orx* gene expression in the hypothalamus in EtOH rats. **p < 0.01, vs. naive; ^##^*p* < 0.01, vs. EtOH_ND_. **(E)** Relative *OrxR1* gene expression in the pPVT in EtOH rats. **p < 0.01, vs. naïve; ^##^*p* < 0.01, vs. EtOH_ND_. **(F)** Relative *OrxR2* gene expression in the pPVT in EtOH rats. *p < 0.05, vs. naive; ^#^*p* < 0.05, vs. EtOH_ND_. *Orx*, *OrxR1*, and* OrxR2* mRNA expression levels were normalized to glyceraldehyde-3-phosphate dehydrogenase (*Gapdh*) and are expressed in relative amounts. *n* = 8 rats/group. Blue symbols indicate male rats. Purple symbols indicate female rats. BSL, baseline; W4, week 4; W5, week 5; W6, week 6; EXT, extinction; SH, sham injection; SIR, stress-induced reinstatement; N, naive; ND, EtOH_ND_; D, EtOH_D_.

**Figure 4:**
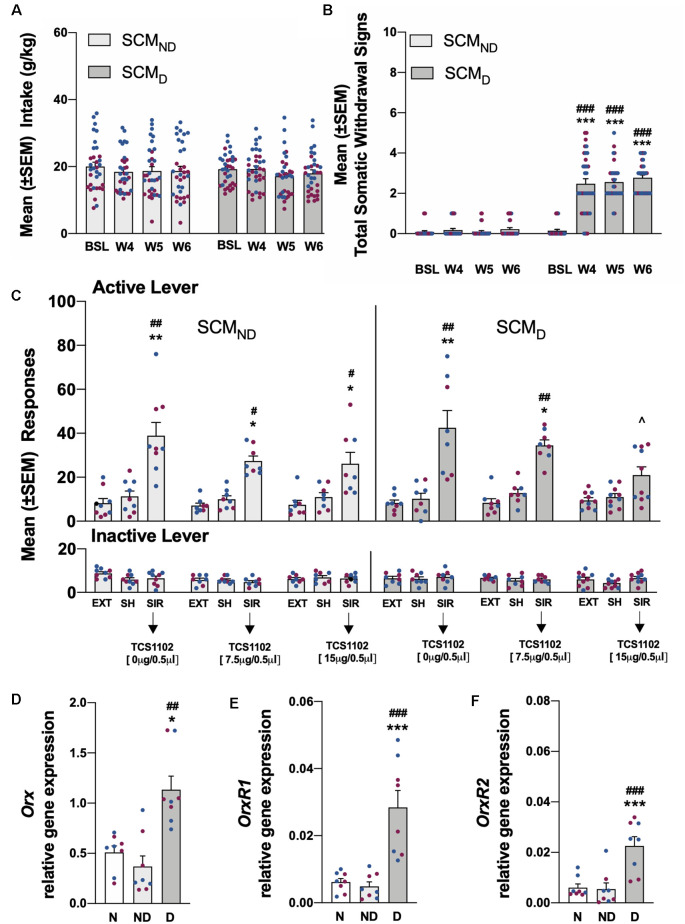
**(A)** Total SCM intake in the SCM_ND_ and SCM_D_ groups during training and the air (SCM_ND_) and CIE vapor procedure (SCM_D_). *n* = 33–34 rats/group. **(B)** Somatic withdrawal signs (WDS) recorded upon the completion of training and during acute abstinence during CIE vapor exposure. ***p < 0.001, vs. baseline; ^###^*p* < 0.001, vs. SCM_ND_. *n* = 33–34 rats/group. **(C)** Effect of TCS1102 injection in the pPVT on footshock stress-induced reinstatement of SCM-seeking behavior. *p < 0.05, **p < 0.01, vs. respective extinction and sham injection; ^∧^*p* < 0.05, vs. respective vehicle; ^#^*p* < 0.05, ^##^*p* < 0.01, vs. respective inactive lever. *n* = 8–10 rats/group. **(D)** Relative *Orx* gene expression in the hypothalamus in SCM rats. *p < 0.05, vs. naive; ^##^*p* < 0.01, vs. SCM_ND_. **(E)** Relative *OrxR1* gene expression in the pPVT in SCM rats. ***p < 0.001, vs. naive; ^###^*p* < 0.001, vs. SCM_ND_. **(F)** Relative *OrxR2* gene expression in the pPVT in SCM rats. ***p < 0.001, vs. naive; ^###^*p* < 0.001, vs. SCM_ND_. *Orx*, *OrxR1*, and* OrxR2* mRNA expression levels were normalized to glyceraldehyde-3-phosphate dehydrogenase (*Gapdh*) and are expressed in relative amounts. *n* = 8 rats/group. Blue symbols indicate male rats. Purple symbols indicate female rats. BSL, baseline; W4, week 4; W5, week 5; W6, week 6; EXT, extinction; SH, sham injection; SIR, stress-induced reinstatement; N, naive; ND, SCM_ND_; D, SCM_D_.

### Ethanol and Sweetened Condensed Milk Self-administration Training (Figure 1)

Ethanol and SCM self-administration were established in daily 30 min sessions on a fixed-ratio 1 (FR1) schedule of reinforcement in standard operant conditioning chambers (29 × 24 × 19.5 cm; Med Associates, St. Albans, VT, USA). Sessions were initiated by the extension of both levers into the operant chamber, and responses on the right active lever resulted in the delivery of 0.1 ml of 10% EtOH (w/v) or SCM diluted 2:1 (v/v) in tap water into a drinking receptacle and the brief illumination of a cue light (0.5 s) above the lever. Responses on the left inactive lever were recorded but had no scheduled consequences. The rats were scored for somatic withdrawal signs (WDS) upon the completion of training (Figure 1).

### PVT Cannulation (Figure 1)

Fourteen days after beginning self-administration training, the rats that were assigned to stress-induced reinstatement testing (*n* = 142) were implanted with a guide cannula (23-gauge, 15 mm, Plastics One, Roanoke, VA, USA) that was aimed at the PVT anterior/posterior, −3.3 mm; medial/lateral, ±2.72 mm from Bregma; dorsal/ventral, −2.96 mm from the dura, 25° angle; (Paxinos and Watson, 1997) and positioned 3.5 mm above the target injection point. The PVT coordinates that were used are within the posterior part of the PVT (i.e., pPVT, the portion of the PVT that receives the most Orx afferents; Kirouac et al., 2005). After 7 days of recovery, the animals resumed self-administration training for an additional 7 days.

### Chronic Intermittent EtOH Vapor Exposure (Figure 1)

After 21 sessions of EtOH or SCM self-administration, half of the rats were made dependent (EtOH_D_or SCM_D_) by chronic intermittent EtOH (CIE) vapor exposure. The other half was exposed only to air (non-dependent groups, EtOH_ND_or SCM_ND_). During 6 weeks of dependence induction, the rats underwent daily cycles of 14 h EtOH vapor ON [blood alcohol levels (BALs) during vapor exposure ranged between 150 and 250 mg%, measured with a blood analyzer (GC-headspace, Agilent Technologies, Santa Clara, CA, USA)] and 10 h OFF and were left undisturbed for 3 weeks except to control BALs (measured during the last 15 min of vapor exposure) and score withdrawal signs (WDS; at 8 h of abstinence) once per week. Behavioral signs of withdrawal were measured by a laboratory assistant who was blind to the experimental conditions using a rating scale that was adapted from an original study by Macey et al. (1996). The scale included ventromedial limb retraction, vocalization (i.e., irritability in response to touch), tail rigidity, abnormal gait, and body tremors. Each sign was given a score of 0–2, based on the following severity: 0 = no sign, 1 = moderate, and 2 = severe. The sum of the five scores (0–10) was used as a quantitative measure of withdrawal severity and to confirm dependence. In this model, rats exhibit somatic and motivational signs of withdrawal (Vendruscolo and Roberts, 2014). Starting at the beginning of the fourth week of CIE vapor exposure, the rats were subjected to 30 min FR1 EtOH or SCM self-administration sessions when acute abstinence occurred (i.e., 8 h after the vapor was turned off when brain and blood alcohol levels are negligible), three times per week (Monday, Wednesday, and Friday). The air-exposed rats underwent the same procedure. During weeks 7 and 8 of CIE vapor exposure, the rats were subjected to daily 30 min extinction sessions when acute abstinence occurred. Extinction sessions were identical to the EtOH or SCM self-administration sessions but with EtOH or SCM withheld. Fifteen minutes before the last extinction session, the rats received a sham injection to habituate them to the microinjection procedure. This involved the insertion of an injector (that was left in place for 2 min) in the guide cannula that extended into the pPVT. After the sham injections, the rats were returned to their home cages for 2 min and then placed in the operant chamber for 15 min. At the end of the 15-min period, both levers in the operant chambers were extended, and the rats were tested under extinction conditions.

### Effects of TCS1102 on the Stress-Induced Reinstatement of EtOH- and SCM-Seeking Behavior (Figure 1)

Twenty-four hours after the sham injection session during acute abstinence, the rats received TCS1102 (Tocris Bioscience, Bristol, UK; 0, 7.5, or 15 μg; Hsiao et al., 2012; Dong et al., 2015) dissolved in 100% dimethylsulfoxide (DMSO; Sigma Aldrich, St. Louis, MO, USA) and then tested for the reinstatement of EtOH and SCM seeking that was induced by footshock stress. The microinjections in the pPVT were performed using a micro-infusion pump (Harvard 22 Syringe Pump, Holliston, MA, USA) and injectors that extended 3.5 mm beyond the guide cannula. The injections were performed at a flow rate of 0.5 μl/min over 1 min. The injectors were left in place for an additional minute to allow for diffusion away from the injector tip. Following the injections, the rats were returned to their home cages for 2 min and then placed in the operant chamber to undergo footshock stress [15 min, variable intermittent electric footshock, 0.5 mA; duration, 0.5 s; mean shock interval, 40 s; range, 10–70 s; (Martin-Fardon et al., 2000; Zhao et al., 2006; Sidhpura et al., 2010)]. Two minutes following the termination of footshock, levers were extended into the chamber, and responses were recorded for 30 min. Each animal was tested with only one dose of TCS1102 or a vehicle according to a between-subjects design. Injection sites were verified, and off-target cannulations were excluded from the study (Figure 2).

### Quantitative Polymerase Chain Reaction (qPCR) Procedure

The rats that were used for the gene expression analysis (*n* = 48) were prepared in parallel and underwent the same behavioral procedure as described above, but they did not undergo pPVT cannulation. Therefore, these rats were neither injected with TCS1102 nor tested for stress-induced reinstatement. Twenty-four hours after the last extinction session (at 8 h of abstinence, at the same time when the effects of TCS1102 on stress-induced reinstatement were tested in the behavioral groups), the rats were deeply anesthetized and decapitated. Their brains were rapidly extracted, frozen in methyl butane, and stored at −80°C. Brains were subsequently dissected into coronal sections, and brain regions of interest were collected with tissue punches. The sampled regions included the hypothalamus (dorsolateral, including the dorsomedial, perifornical, and lateral hypothalamus; range concerning Bregma: −2.56 to −4.16 mm; Paxinos and Watson, 1997) and the pPVT (range concerning Bregma: −2.80 to −3.80 mm). Brain punches were frozen on dry ice and stored at −80°C. Total RNA was isolated and purified using an RNA extraction kit (RNA Clean and Concentrator-5, Zymo Research, Irvine, CA, USA). RNA concentration was measured using a NanoDrop 2000c spectrophotometer (Thermo Fisher Scientific, Waltham, MA, USA). Total RNA was reverse transcribed into complementary DNA (cDNA) *via* 5× mix, iScript, Reverse Transcription, Supermix for RT-qPCR using a CFX 384 Real-Time System (Bio-Rad, Hercules, CA, USA). The cDNA templates were amplified using SYBR, iTaq Universal SYBR, and Green Supermix in the CFX 384 Real-Time System (Bio-Rad, Hercules, CA, USA). The primer sequences of *Orx* *(prepro-Orx)* antisense oligonucleotides were 5′-GCC GTC TCT ACG AAC TGT TG-3′ and 5′-CGA GGA GAG GGG AAA GTT AG-3′. The antisense oligonucleotide primer sequences were 5′-CCC TCA ACT CCA GTC CTA GC-3′ and 5′-CAG GGA GGG CCT ATA ATT GA-3′ for *OrxR1* and 5′-CCA TGT TGT TGG GGT GCT TA-3′ and 5′-TCC CCC TCT CAT AAA CTT GG-3′ for* OrxR2*. The primer sequences of the housekeeping gene glyceraldehyde-3-phosphate dehydrogenase (*Gapdh*) were 5′-CAA GGC TGT GGG CAA GGT CA-3′ and 5′-GGT TTC TCC AGG CGG CAT GT-3′ (Jöhren et al., 2001). The relative expression of mRNA was calculated using the comparative Ct method. All data were standardized with *Gapdh* as the endogenous reference gene. Relative expression of different gene transcripts was calculated by the ΔCq method and converted to a relative expression ratio (2^−ΔCq^) for the statistical analysis (Livak and Schmittgen, 2001).

### Statistical Analysis

Ethanol and SCM self-administration data were analyzed using two-way repeated-measures analysis of variance (ANOVA), with time (i.e., baseline, weeks 4–6) and dependence as factors. Withdrawal score values were log10 transformed for the statistical analysis and back-transformed for a graphical representation (Matzeu et al., 2018) and analyzed using two-way ANOVA, with time and dependence as factors. Stress-induced reinstatement was analyzed using a mixed three-way ANOVA, with treatment (i.e., responses during the extinction, sham, and reinstatement test following the TCS1102 injection), dependence (i.e., EtOH_ND_, vs. EtOH_D_/SCM_D_ vs. SCM_ND_), and lever (i.e., active vs. inactive) as factors. Significant main effects or interactions in the ANOVAs were followed by the Tukey *post hoc* test. Relative gene expression data were analyzed using one-way ANOVA, followed by the Tukey *post hoc* test. All of the results are expressed as mean ± SEM. Values of *p* < 0.05 were considered statistically significant. The statistical analysis was performed using GraphPad Prism 8 software.

## Results

Nineteen animals were excluded from the study (three that never acquired self-administration, five because of health complications, and 11 because of cannula misplacement), thus reducing the number of animals to 161 (EtOH_ND_: *n* = 34 for behavior and *n* = 8 for qPCR; EtOH_D_: *n* = 28 for behavior and *n* = 8 for qPCR; SCM_ND_: *n* = 25 for behavior and *n* = 8 for qPCR; SCM_D_: *n* = 26 for behavior and *n* = 8 for qPCR; naive: *n* = 16 for qPCR).

### Ethanol Self-administration and Stress-Induced Reinstatement

Before CIE vapor or air exposure, the rats were divided into two subgroups to obtain a similar EtOH self-administration baseline (Figure 3A) based on the last 3 days of training. During weeks 4–6 of CIE vapor exposure, compared with pre-CIE vapor exposure and the EtOH_ND_ group, EtOH_D_ animals escalated their EtOH intake [calculated by averaging the measures that were obtained Monday, Wednesday, and Friday during weeks 4–6 of the CIE procedure; *p* < 0.001, Tukey *post hoc* test following two-way ANOVA: dependence (EtOH_ND_ vs. EtOH_D_), *F*_(1,76)_ = 29.06, *p* < 0.001; time, *F*_(3,228)_ = 41.85, *p* < 0.001; dependence × time interaction, *F*_(3,228)_ = 22.00, *p* < 0.001; Figure 3A]. During weeks 4–6 of CIE vapor exposure, somatic withdrawal signs were measured at 8 h of abstinence. EtOH_D_ animals exhibited significantly higher somatic withdrawal signs compared with withdrawal signs that were measured at the end of EtOH self-administration training and compared with the EtOH_ND_ group [*p* < 0.001, Tukey *post hoc* test following two-way ANOVA: dependence (EtOH_ND_ vs. EtOH_D_), *F*_(1,336)_ = 130.20, *p* < 0.001; time, *F*_(3,228)_ = 44.46, *p* < 0.001; dependence × time interaction, *F*_(3,228)_ = 39.52, *p* < 0.001; Figure 3B]. Compared with extinction and the sham injection, footshock elicited the significant reinstatement of EtOH-seeking behavior in both the EtOH_ND_ and EtOH_D_ groups [*p* < 0.05, Tukey *post hoc* test following three-way ANOVA: dependence (EtOH_ND_ vs. EtOH_D_), *F*_(1,336)_ = 0.13, *p* > 0.05; treatment (extinction, sham, TCS1102), *F*_(8,336)_ = 10.72, *p* < 0.001; lever (active vs. inactive), *F*_(1,336)_ = 89.0, *p* < 0.001; dependence × treatment interaction, *F*_(8,336)_ = 1.42, *p* > 0.05; lever × treatment interaction, *F*_(8,336)_ = 10.75, *p* < 0.001; dependence × lever interaction, *F*_(8,336)_ = 0.01, *p* > 0.05; dependence × treatment × lever interaction, *F*_(8,336)_ = 1.25, *p* > 0.05; Figure 3C]. The injection of TCS1102 in the pPVT reduced the footshock-induced reinstatement of EtOH-seeking behavior only in EtOH_D_ rats, with a significant effect at the 15-μg dose (*p* < 0.05, Tukey *post hoc* test; Figure 3C). Responses at the inactive lever remained low and unaffected throughout the experiment (Figure 3C).

### Sweetened Condensed Milk Self-administration and the Stress-Induced Reinstatement

Compared with pre-CIE vapor exposure and the SCM_ND_ group, SCM self-administration remained stable and unchanged in SCM_D_ rats [two-way ANOVA: dependence (SCM_ND_ vs. SCM_D_), *F*_(1,65)_ = 0.11, *p* > 0.05; time, *F*_(3,195)_ = 2.34, *p* > 0.05; dependence × time interaction, *F*_(3,195)_ = 0.96, *p* > 0.05; Figure 4A]. During weeks 4–6 of CIE vapor exposure, somatic withdrawal signs were measured at 8 h of abstinence. SCM_D_ rats exhibited significantly higher somatic withdrawal signs compared with withdrawal signs that were measured at the end of SCM self-administration training and compared with the SCM_ND_ group [*p* < 0.001, Tukey *post hoc* test following two-way ANOVA: dependence (SCM_ND_ vs. SCM_D_), *F*_(1,65)_ = 295.7, *p* < 0.001; time, *F*_(3,195)_ = 62.65, *p* < 0.001; dependence × time interaction, *F*_(3,195)_ = 54.80, *p* < 0.001; Figure 4B]. Footshock stress induced the reinstatement of SCM seeking in both the SCM_ND_ and SCM_D_ groups [*p* < 0.05, Tukey *post hoc* test following three-way ANOVA: dependence (SCM_ND_ vs. SCM_D_), *F*_(1,235)_ = 0.04, *p* > 0.05; treatment (extinction, sham, TCS1102), *F*_(8,235)_ = 13.78, *p* < 0.001; lever (active vs. inactive), *F*_(1,235)_ = 79.19, *p* < 0.001; dependence × treatment interaction, *F*_(8,235)_ = 0.33, *p* > 0.05; lever × treatment interaction, *F*_(8,235)_ = 13.64, *p* < 0.001; dependence × lever interaction, *F*_(8,235)_ = 0.14, *p* > 0.05; dependence × treatment × lever interaction, *F*_(8,235)_ = 0.27, *p* > 0.05; Figure 4C]. The injection of TCS1102 in the pPVT reduced the footshock stress-induced reinstatement of SCM-seeking behavior only in SCM_D_ rats, with a significant effect at the 15-μg dose (*p* < 0.05, Tukey *post hoc* test; Figure 4C), similar to the EtOH_D_ group. Responses at the inactive lever remained low and unaffected (Figure 4C).

### Relative Gene Expression Analysis of Orx in the Hypothalamus and OrxR1/2 Expression in the pPVT

Relative *Orx* gene expression in the hypothalamus significantly increased in rats in the EtOH_D_ and SCM_D_ groups compared with naive and nondependent rats (EtOH: *p* < 0.01, Tukey *post* *hoc* test following one-way ANOVA, *F*_(2,21)_ = 10.52, *p* < 0.001; SCM: *p* < 0.05, Tukey *post hoc* test following one-way ANOVA, *F*_(2,21)_ = 7.06, *p* < 0.01). Relative *Orx* gene expression in the EtOH_ND_ and SCM_ND_ groups was identical to the naive group (Figures 3D, 4D). In the EtOH_D_ and SCM_D_ groups, the relative gene expression of *OrxR1* and *OrxR2* in the pPVT was significantly higher than in the naive and non-dependent groups (*OrxR1* for EtOH: *p* < 0.01, Tukey *post hoc* test following one-way ANOVA, *F*_(2,21)_ = 10.90, *p* < 0.001; *OrxR1* for SCM: *p* < 0.001, Tukey *post hoc* test following one-way ANOVA, *F*_(2,21)_ = 18.56, *p* < 0.001; *OrxR2* for EtOH: *p* < 0.05, Tukey *post hoc* test following one-way ANOVA, *F*_(2,21)_ = 4.76, *p* < 0.05; *OrxR2* for SCM: *p* < 0.001, Tukey *post hoc* test following one-way ANOVA, *F*_(2,21)_ = 13.50, *p* < 0.001). In the EtOH_ND_ and SCM_ND_ groups, relative *OrxR1* and *OrxR2* gene expression were similar to relative *OrxR1* and *OrxR2* gene expression in the naive group (Figures 3E,F, 4E,F).

## Discussion

In the present study, the influence of EtOH dependence on the self-administration of EtOH or a highly palatable food (SCM) was evaluated. As reported previously (e.g., O’Dell et al., 2004; Vendruscolo and Roberts, 2014; Matzeu et al., 2018), EtOH_D_ rats exhibited an increase (i.e., escalation) in EtOH self-administration during dependence, whereas SCM self-administration was unaffected, suggesting that the intake of highly palatable food, in contrast to EtOH, does not alleviate negative withdrawal states (for review, see Koob, 2014). Furthermore, we observed a pivotal role for Orx inputs to the pPVT in the stress-induced reinstatement of reward-seeking behavior in EtOH-dependent rats. Footstock stress-induced the reinstatement of reward-seeking behavior in all groups, but an intra-pPVT injection of the dual Orx receptor antagonist TCS1102 reduced reinstatement only in dependent rats (i.e., EtOH_D_and SCM_D_ groups), with no effect in nondependent rats (i.e., EtOH_ND_ and SCM_ND_ groups). The relative gene expression analysis revealed that *Orx* mRNA expression in the hypothalamus and *OrxR1* and *OrxR2* mRNA expression in the pPVT significantly increased in the EtOH_D_ and SCM_D_ groups compared with the naive and non-dependent groups. Overall, the behavioral and molecular data are the first to demonstrate the maladaptive recruitment of Orx inputs to the pPVT by EtOH dependence.

People who suffer from AUD are inclined to increase their EtOH consumption to relieve or avoid withdrawal symptoms (Peer et al., 2013). Similarly, in preclinical studies, EtOH-dependent rats exhibited traits of EtOH dependence that were characterized by somatic and motivational withdrawal symptoms that typically appeared after 6–8 h of abstinence from EtOH, and EtOH self-administration (escalation) significantly increases when EtOH is made available again (Roberts et al., 1996; O’Dell et al., 2004; Vendruscolo and Roberts, 2014; Matzeu et al., 2018). Consistent with these earlier observations, we found an increase in EtOH self-administration in EtOH_D_ rats during weeks 4–6 of CIE vapor exposure, whereas SCM intake was unaffected in SCM_D_ rats. These findings demonstrate that the consumption of highly palatable food, in contrast to EtOH, was unable to relieve negative states that are associated with acute EtOH withdrawal. Drugs of abuse usurp neurocircuitry that controls food intake (Volkow et al., 2012, 2013; Tomasi and Volkow, 2013) and it has been described that palatable food and EtOH intake is controlled by common neuronal substrates (Barson et al., 2011; Barson and Leibowitz, 2016). Both clinical and preclinical studies have shown that moderate EtOH consumption increases palatable food intake (Schrieks et al., 2015; Cummings et al., 2020), but heavy EtOH consumption either decreases or does not change palatable food intake (Cummings et al., 2020). These previous findings were confirmed by the present results, showing no changes in SCM consumption during EtOH dependence (i.e., heavy EtOH exposure). The reason why EtOH dependence did not influence SCM consumption is unclear. However, as suggested by others (for review, see Cummings et al., 2020), the dose-dependent effects of EtOH on palatable food intake could overlap with the biphasic effect of EtOH (i.e., increase in food intake at a low dose of EtOH and no change or a decrease in food intake at a high dose of EtOH). If low-dose EtOH can increase the motivation for palatable food, the withdrawal state that is induced by chronic exposure to high doses of EtOH (i.e., CIE vapor exposure) can be alleviated only by a voluntary increase in EtOH self-administration (escalation) but not SCM self-administration (no escalation). An alternative explanation for the absence of an increase in SCM self-administration in SCM_D_ rats could be attributable to the schedule of reinforcement that was used. Under the present experimental conditions, the level of responding for SCM was already substantially high under basal conditions. This observation suggests that the SCM group of rats might have already reached a plateau of intake before EtOH dependence induction, thus dramatically limiting the possibility of measuring any further increase in SCM self-administration. Nonetheless, further studies are needed to support or refute this hypothesis.

Although footstock stress-induced reinstatement in all groups in the present study, the intra-pPVT injection of TCS1102 reduced reinstatement only in the EtOH_D_ and SCM_D_ groups, with no effect in the EtOH_ND_ and SCM_ND_ groups. Before discussing the implications of these findings, a potential limitation needs to be mentioned. A possible behavioral confound following the TCS1102 injection in the pPVT could be the close position of the pPVT to the third ventricle and thus the possibility that TCS1102 diffused to the ventricles and exerted nonspecific actions at other brain regions beyond the pPVT. However, the accuracy of the injections (depicted in Figure 2), together with our earlier studies that used a similar approach and found: (1) a selective effect of transient inactivation of the pPVT in preventing cocaine conditioned reinstatement (Matzeu et al., 2015); and (2) selective blockade of the reinstating effect of intra-pPVT OrxA administration by co-administering the OrxR2 antagonist TCSOX229 (Matzeu et al., 2016) or dynorphin (Matzeu et al., 2018), strongly argue against this possibility. The Orx system is well known to play an important role in reward-seeking behavior, especially when the motivation for the reward is high (Borgland et al., 2009; Moorman and Aston-Jones, 2009; España et al., 2010; Hollander et al., 2012; Mahler et al., 2014; Bentzley and Aston-Jones, 2015; Lopez et al., 2016). Consistent with this observation, several studies reported that the effect of OrxR antagonists is more pronounced in animals with high motivation for EtOH (Anderson et al., 2018), suggesting a role for the Orx system in the exacerbation of EtOH seeking and drinking that are typically observed with EtOH dependence. For example, systemic SB334867 administration produced more robust decreases in EtOH self-administration and reinstatement in selectively bred alcohol-preferring (P) rats (Lawrence et al., 2006; Dhaher et al., 2010; Anderson et al., 2014). SB334867 also decreased EtOH drinking and preference to a greater extent in rats with a high preference for EtOH but only weakly in rats with a low preference for EtOH (Moorman and Aston-Jones, 2009; Moorman et al., 2017). The blockade of OrxR1 selectively decreased the escalation of drinking in EtOH-dependent mice, without altering lower levels of EtOH intake in non-dependent mice (Lopez et al., 2016) and was more effective in reducing compulsive-like EtOH drinking in quinine-resistant mice (Lei et al., 2016a,b). Finally, the dual OrxR antagonist almorexant decreased the breakpoint for EtOH seeking in a progressive-ratio schedule in rats that were genetically predisposed to EtOH preference (Anderson et al., 2014). Collectively, these previous studies and the present findings suggest that the extent to which the Orx system is recruited is linked to greater motivation that is induced by EtOH dependence in EtOH_D_ and SCM_D_ rats. In the EtOH_D_ and SCM_D_ groups, dysregulation or hyperactivation of the Orx system could produce pathological EtOH and SCM seeking. In fact, following intra-pPVT vehicle injections, the magnitude of stress-induced reinstatement was virtually identical in all groups (dependent and nondependent). However, the observation that TCS1102 prevented stress-induced reinstatement only in EtOH-dependent rats (EtOH_D_ and SCM_D_ groups) is indicative of a common mechanism that mediates the stress-induced reinstatement of EtOH seeking or palatable food-seeking following EtOH dependence, strongly suggesting that CIE vapor exposure induces neuroadaptations that can be revealed by pharmacological manipulations. If this hypothesis is correct, then antagonizing the Orx system could be particularly valuable because it would help prevent EtOH craving and relapse in individuals with AUD and perhaps be beneficial for treating maladaptive behavior toward conventional reinforcers in individuals with a history of EtOH dependence (e.g., Schuckit et al., 1996; Sinha and O’Malley, 2000; Munn-Chernoff et al., 2013). Such a possibility will need further investigation.

The present study showed that the blockade of both OrxRs in the pPVT with the dual OrxR antagonist TCS1102 prevented stress-induced reinstatement only in EtOH-dependent rats. The PVT has been proposed to be part of the neurocircuitry that regulates drug-seeking (Martin-Fardon and Boutrel, 2012) and has been shown to participate in hedonic feeding (Choi et al., 2012). Indeed, the PVT is involved in cocaine and EtOH seeking and reinstatement (Dayas et al., 2008; Hamlin et al., 2009; James et al., 2010, 2011; Matzeu et al., 2016, 2018). The anterior PVT has also been shown to play an important role in regulating EtOH drinking (Barson et al., 2015, 2017). CIE exposure or EtOH intoxication reduces Fos expression in the PVT, and Fos expression markedly increases during acute withdrawal following CIE exposure (Smith et al., 2019). The present results extend our understanding of the participation of Orx inputs in the pPVT in the mediation of stress-induced EtOH- and SCM-seeking behavior after a history of EtOH dependence. The PVT receives dense Orx projections (Peyron et al., 1998; Baldo et al., 2003; Kirouac et al., 2005; Hsu and Price, 2009) and is implicated in behavioral responses to acute and chronic stressors (Hsu et al., 2014) and appetitive and aversive behaviors (Hsu et al., 2014; Kirouac, 2015; Millan et al., 2017). Barson et al. (2015) found that an injection of OrxA and OrxB peptides in the anterior PVT but not pPVT increased EtOH consumption, an effect that was reversed by an injection of the Orx2R antagonist TCSOX229 (Barson et al., 2015, 2017), supporting the hypothesis that the anterior PVT plays a role in mediating EtOH drinking *via* OrxR2 signaling. The lack of an effect on EtOH drinking when injections occurred in the pPVT suggests regional specialization of the PVT, in which the anterior PVT is most likely engaged during EtOH and perhaps SCM consumption, whereas the pPVT (present study) mediates the reinstatement of reward-seeking behavior (EtOH or SCM) that is induced by stress in rats with a history of EtOH dependence. Further studies are needed to elucidate the possible specialization of the PVT subdivisions.

The lack of effect of TCS1102 in EtOH nondependent animals suggests that the stress-induced reinstatement of reward-seeking behavior in these rats might not depend on pPVT Orx transmission but perhaps depend on other mechanisms. However, a large body of evidence demonstrates the involvement of the Orx system and PVT in feeding (Sutcliffe and de Lecea, 2000; Mieda and Yanagisawa, 2002; Kelley et al., 2005; de Lecea, 2012), arousal, stress, anxiety (Bhatnagar and Dallman, 1998; Van der Werf et al., 2002; Kelley et al., 2005; Parsons et al., 2006; Berridge et al., 2010; Hsu et al., 2014), and reward-motivated behavior (Harris et al., 2005; Dayas et al., 2008; Martin-Fardon et al., 2010, 2018; Jupp et al., 2011b; Sakurai and Mieda, 2011; Hsu et al., 2014), strongly arguing against this possibility. Another possibility is that at the dose range used, TCS1102 has no affect in EtOH_ND_ and SCM_ND_ rats because their Orx/OrxR system is not compromised. Supporting this possibility is a recent study that showed that OrxR2 blockade with TCSOX229 in the pPVT reduced cue-induced food-seeking behavior in hungry rats (i.e., a stressful condition; Meffre et al., 2019). Overall, these findings confirm that Orx transmission in the pPVT plays an important role in reward-seeking behavior that is induced by cues or stress, especially when the motivation for the reward is high or when the Orx system is compromised, such as by food restriction (Diano et al., 2003; Horvath and Gao, 2005; Iwasa et al., 2015) or EtOH dependence (present study).

At the time of reinstatement testing in the present study, significant increases in *Orx* mRNA expression in the hypothalamus and *OrxR1* and *OrxR2* mRNA expression in the pPVT in EtOH_D_ and SCM_D_ rats were observed. In the lateral hypothalamus, an increase in *Orx* mRNA expression was observed during morphine (Zhou et al., 2006) and cocaine (Zhou et al., 2008) withdrawal. Particularly relevant is the observation that chronic EtOH consumption increases *Orx* mRNA expression in the lateral hypothalamus in EtOH-preferring rats (Lawrence et al., 2006) and non-genetically selected EtOH-preferring rats and *Orx2R* mRNA expression in the anterior PVT (Barson et al., 2015). Similarly, oral EtOH administration by gavage increased the double-labeling of Fos with Orx2R in the anterior PVT (Barson et al., 2015). We propose that Orx overexpression that is induced by chronic EtOH administration and the lack of an ability to reduce OrxR expression and activity in response to chronic EtOH administration might make rats vulnerable to EtOH overconsumption and craving. In fact, under normal physiological conditions, Orx synthesis triggers an adaptive reduction of OrxRs in the lateral hypothalamus that exerts negative feedback on enhanced Orx activity (Alcaraz-Iborra et al., 2014). Following EtOH dependence, this regulatory balance between Orx peptides and receptors might be compromised. This possibility is supported by the finding that acute EtOH increases the density of Orx neurons in the lateral hypothalamus and the observation that repetitive sessions of EtOH binge-like drinking failed to significantly reduce mRNA *OrxR1* expression (Morganstern et al., 2010). Perhaps EtOH dependence-induced maladaptation leads to the exacerbation of Orx signaling in the pPVT. This maladaptation was behaviorally revealed in the present study by the pharmacological effects of TCS1102 that antagonized stress-induced reinstatement in EtOH_D_ and SCM_D_ rats and the observation of the upregulation of *Orx* and *OrxRs* gene expression. One limitation of the present qPCR analysis could be that *Orx* and *OrxRs* gene expression was measured at acute abstinence, without subjecting the rats to stress. Although investigating how stress itself can cause additional changes in the Orx system would be of interest, the rationale for processing the tissue under the present conditions was to obtain a snapshot of the Orx/OrxR system at the time when the effects of TCS1102 on stress-induced reinstatement were tested in the behavioral groups (i.e., 24 h after the last extinction session at 8 h of abstinence).

Stress is a major cause of relapse in patients who suffer from AUD (Breese et al., 2005; Sinha, 2007). The present results demonstrated the maladaptive recruitment of Orx transmission in the pPVT by EtOH dependence, reflected by changes in transcription factors in the Orx/OrxR system. Dual OrxR antagonist administration in the pPVT prevented the stress-induced reinstatement of EtOH- and SCM-seeking behavior in rats with a history of EtOH dependence and molecular changes were induced by CIE vapor exposure in both EtOH and SCM self-administering rats. Overall, these findings suggest that targeting OrxRs could have beneficial effects on preventing EtOH craving and relapse and possibly preventing more general maladaptive behavior toward conventional reinforcers.

## Data Availability Statement

The raw data supporting the conclusions of this article will be made available by the authors, without undue reservation.

## Ethics Statement

All of the procedures were conducted in strict adherence to the National Institutes of Health Guide for the Care and Use of Laboratory Animals and were approved by the Institutional Animal Care and Use Committee of The Scripps Research Institute.

## Author Contributions

AM and RM-F participated in the study concept and design. AM performed the experiments, undertook the statistical analysis, interpreted the findings, and drafted the manuscript. All authors contributed to the article and approved the submitted version.

## Conflict of Interest

The authors declare that the research was conducted in the absence of any commercial or financial relationships that could be construed as a potential conflict of interest.
